# Sperm and offspring production in a nonobstructive azoospermia mouse model via testicular mRNA delivery using lipid nanoparticles

**DOI:** 10.1073/pnas.2516573122

**Published:** 2025-10-13

**Authors:** Daisuke Mashiko, Chihiro Emori, Yuki Hatanaka, Daisuke Motooka, Chen Pan, Yuki Kaneda, Martin M. Matzuk, Masahito Ikawa

**Affiliations:** ^a^Department of Experimental Genome Research, Research Institute for Microbial Diseases, The University of Osaka, Suita, Osaka 565-0871, Japan; ^b^Next Generation Sequencing Core Facility, Research Institute for Microbial Diseases, The University of Osaka, Suita, Osaka 565-0871, Japan; ^c^Center for Drug Discovery, Department of Pathology & Immunology, Baylor College of Medicine, Houston, TX 77030; ^d^Center for Advanced Modalities and Drug Delivery System, The University of Osaka, Osaka 565-0871, Japan; ^e^Center for Infectious Disease Education and Research, The University of Osaka, Osaka 565-0871, Japan; ^f^The Institute of Medical Science, The University of Tokyo, Minato-ku, Tokyo 108-8639, Japan

**Keywords:** germline therapy, LNP therapy, sterility, azoospermia, lipid nanoparticle

## Abstract

Microsurgical testicular sperm extraction (microTESE) with intracytoplasmic sperm injection (ICSI) represents the current standard treatment for nonobstructive azoospermia (NOA). However, cures remain unavailable for NOA patients lacking retrievable haploid cells. mRNA supplementation could be a potential treatment for genetic defects leading to impaired spermatogenesis. Lipid nanoparticles (LNPs) have emerged as mRNA delivery vehicles with minimal risk of genome integration; however, their ability to selectively deliver mRNA to specific cell types remains limited. To overcome this, microRNA (miRNA) target sequences were incorporated into mRNA constructs to restrict expression specifically to germ cells. Using pyruvate dehydrogenase E1 subunit alpha 2 (PDHA2) knockout mice as an NOA model with meiotic arrest, we demonstrate that LNP-mediated delivery of *Pdha2* mRNA enables the resumption and completion of meiosis, restores sperm production, and facilitates the generation of healthy fertile offspring via ICSI. Whole-genome sequencing of the offspring confirmed the absence of large-scale genomic abnormalities. Our results provide proof of concept for a safe and effective chemically synthesized LNP-based mRNA therapy with miRNA-regulated germ cell specificity, offering a promising therapeutic approach to treating male infertility caused by spermatogenesis arrest.

## Significance

Nonobstructive azoospermia (NOA) caused by genetic defects in meiotic genes remains untreatable, as no curative therapies exist for patients lacking male haploid germ cells. Here, we demonstrate that lipid nanoparticle (LNP)-mediated mRNA delivery targeting the testis-specific gene *Pdha2* restores spermatogenesis in this NOA mouse model. This intervention enabled the production of viable offspring via intracytoplasmic sperm injection. Unlike viral vectors, LNPs provide a safe, nonintegrating strategy for transient gene expression in testicular tissue. Our findings establish a foundation for mRNA-based gene restoration therapy for genetically defined NOA and offer a therapeutic concept for treating human male infertility of genetic origin.

Approximately one in five couples worldwide faces infertility (1), with male factors responsible for nearly half of these cases (2, 3). Azoospermia, the complete absence of spermatozoa in the ejaculate, affects 10 to 15% of infertile men (4). Approximately 40% of azoospermia cases are obstructive azoospermia, which is often amenable to conventional surgical testicular sperm extraction (TESE) followed by intracytoplasmic sperm injection (ICSI). In contrast, nonobstructive azoospermia (NOA), resulting from impaired spermatogenesis, is significantly more difficult to treat. Because NOA patients may have focal areas of sperm production within the testis (5), microscopic testicular sperm extraction (microTESE) is currently employed in NOA cases. Despite these efforts, the sperm retrieval rate remains limited (30 to 50%). This rate drops significantly in patients with mutations in meiosis-related genes, which cause early spermatogenic arrest and result in the absence of retrievable spermatozoa (6). Numerous NOA-associated genes have been identified through genomic studies, and diagnostic panels are now employed to estimate the likelihood of successful sperm retrieval. However, when meiotic gene mutations are detected, current medical interventions offer no effective treatment. Although genetic diagnosis can help avoid unnecessary surgery, it simultaneously underscores the absence of therapeutic options for such patients.

Using animal NOA models, viral vectors have demonstrated therapeutic restoration potential in genetic male infertility (7, 8). Adenoviral and lentiviral vectors predominantly infect Sertoli (somatic) cells in vivo, and their application has been shown to restore spermatogenesis in *Sl*/*Sl^d^* mutant mice, a model of NOA caused by Sertoli cell defects. Further, these viral vectors can efficiently transduce spermatogonial stem cells (SSCs) in vitro, and thus the transduced cells complete spermatogenesis when transplanted back into the testis (9, 10). Recently, adeno-associated viral (AAV) and Sendai viral vectors have been reported to transduce SSCs even in vivo (11, 12); AAV and Sendai viral vectors exhibit a lower risk of genomic integration, making them potentially useful for gene complementation strategies. Nonetheless, the production of viral vectors typically requires packaging in host cells, raising concerns about contamination with cellular components, including host and viral DNA. The risk of germline transgenesis needs to be eliminated for clinical applications. Therefore, we focused on lipid nanoparticles (LNPs), which can be chemically synthesized without the use of living cells and can deliver noninfectious mRNA directly to cells (13), thereby enabling transient expression with minimal risk of genomic integration.

In this study, we evaluated the feasibility of LNP-mediated mRNA delivery into testicular cells and further applications for the treatment of NOA. As a disease model, we used *Pdha2* knockout (KO) mice (14, 15), which exhibit meiotic arrest due to impaired pyruvate dehydrogenase (PDH) complex structure. *Pdha2* encodes the testis-specific E1α subunit of the PDH complex, and its mRNA expression peaks in pachytene spermatocytes (14). Notably, pathogenic variants in human *PDHA2* have been identified in NOA patients (6, 15), underscoring the clinical relevance of this gene. We demonstrate that LNPs can deliver mRNAs into both spermatogenic cells and Sertoli cells in the testis and restore spermatogenesis in *Pdha2*-deficient mice. LNP-mediated mRNA delivery opens a new era for the treatment of male infertility in the clinic.

## Results

### LNP Delivery Into Testicular Cells.

**Fig. 1.**
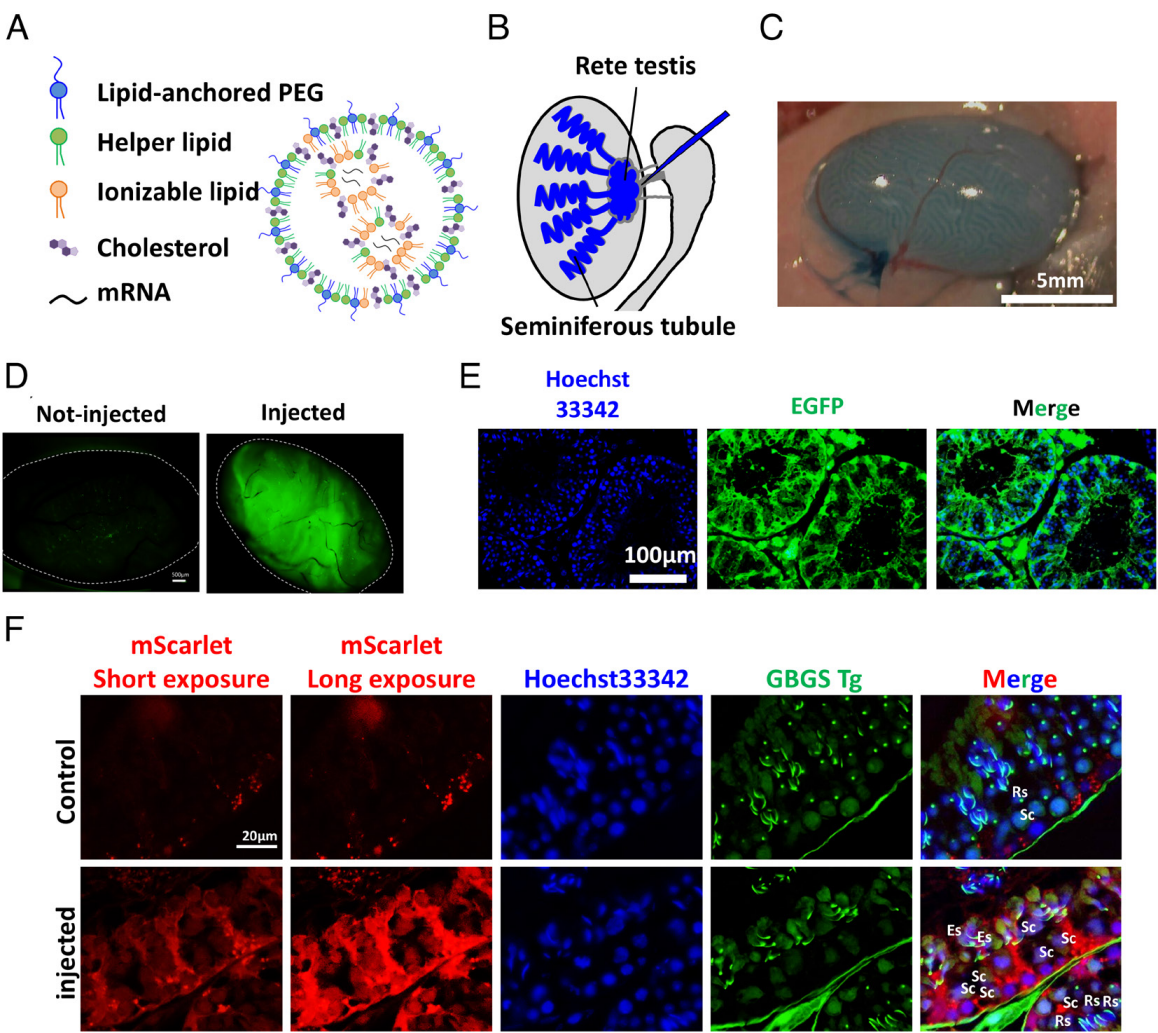
mRNA delivery into testicular cells via LNP. (*A*) Schematic illustration of LNPs. (*B*) Schematic illustration of rete testis injection. (*C*) Testis after *EGFP* mRNA injection via LNP. (*D*) Fluorescence image of the whole testis after LNP-*EGFP* injection. (*E*) Fluorescence image of testis sections after LNP-*EGFP* injection. (*F*) Fluorescence image of testis sections after LNP-*mScarlet* injection in GBGS mice, in which the EGFP signal first appears in the cytoplasm of spermatocytes and later localizes into the acrosome (18). Sc: Spermatocyte, Rs: Round spermatid, Es: Elongated spermatid.

To introduce mRNA into testicular cells, we utilized LNPs (Fig. 1*A*). There are two primary methods for microinjecting reagents into the testis: injection into the interstitial space between seminiferous tubules through the tunica albuginea or injection into the luminal space of seminiferous tubules through the rete testis. While AAVs have been reported to traverse from the interstitial space into the luminal space of seminiferous tubules through the basement membrane and blood-testis barrier (BTB) (11), LNPs have not been reported to do so. Furthermore, LNP-based methodologies for effectively crossing the blood–brain barrier, which shares characteristics with the BTB, are still under investigation (16). Therefore, we selected the rete testis injection (17) as the route for the direct delivery of LNPs into the luminal space of seminiferous tubules (Fig. 1*B*).

LNPs containing mRNA (100 ng/µL) were prepared for injection. Approximately 15 µL of LNP solution containing 0.04% trypan blue was injected into the seminiferous tubules via the rete testis through the efferent duct (Fig. 1*C*). One day after injection of *EGFP* mRNA-containing LNPs (LNP-*EGFP*), fluorescence was observed throughout the seminiferous tubules (Fig. 1*D*). To further analyze LNP distribution, plastic sections of the testes were prepared (Fig. 1*E*). EGFP fluorescence in the injected testis exhibited a radial pattern from the basal compartment to the lumen, consistent with Sertoli cell expression. Examination of entire sections confirmed LNP uptake in 54.9% of the seminiferous tubules (124/226; see *SI Appendix,* Fig. S1).

To determine the duration of protein expression from mRNA delivered via LNPs, we analyzed the seminiferous tubules at 1, 3, 5, and 7 d after injection. Due to the challenges associated with longitudinal in vivo live imaging, testes were collected from different mice, embedded in plastic, and observed under identical gain and exposure conditions. EGFP signals were detected on day 1 and gradually diminished by days 3 and 5, but they became undetectable by day 7 (*SI Appendix,* Fig. S2). These findings indicate that protein translated from exogenous mRNA persists for at least 5 d.

### LNP Delivery Into Spermatogenic Cells.

To examine whether spermatogenic cells internalize LNPs, we first stained the testis injected with LNP-*EGFP* using anti-DDX4 and found spermatogonia also expressed EGFP besides Sertoli cells (*SI Appendix,* Fig. S3). To further follow the differentiation stages of spermatogenic cells, we used GBGS Tg mice expressing Acr3-EGFP (19), a fusion protein targeting the acrosome, as the recipient. In GBGS Tg mice, spermatogenic cells were identified based on the EGFP signal, which first appears in the cytoplasm of spermatocytes and later localizes to the acrosome. When we injected mScarlet (18) mRNA-containing LNPs into the testes of GBGS mice, the mScarlet fluorescence was observed in spermatocytes and spermatids (Fig. 1*F*).

**Fig. 2.**
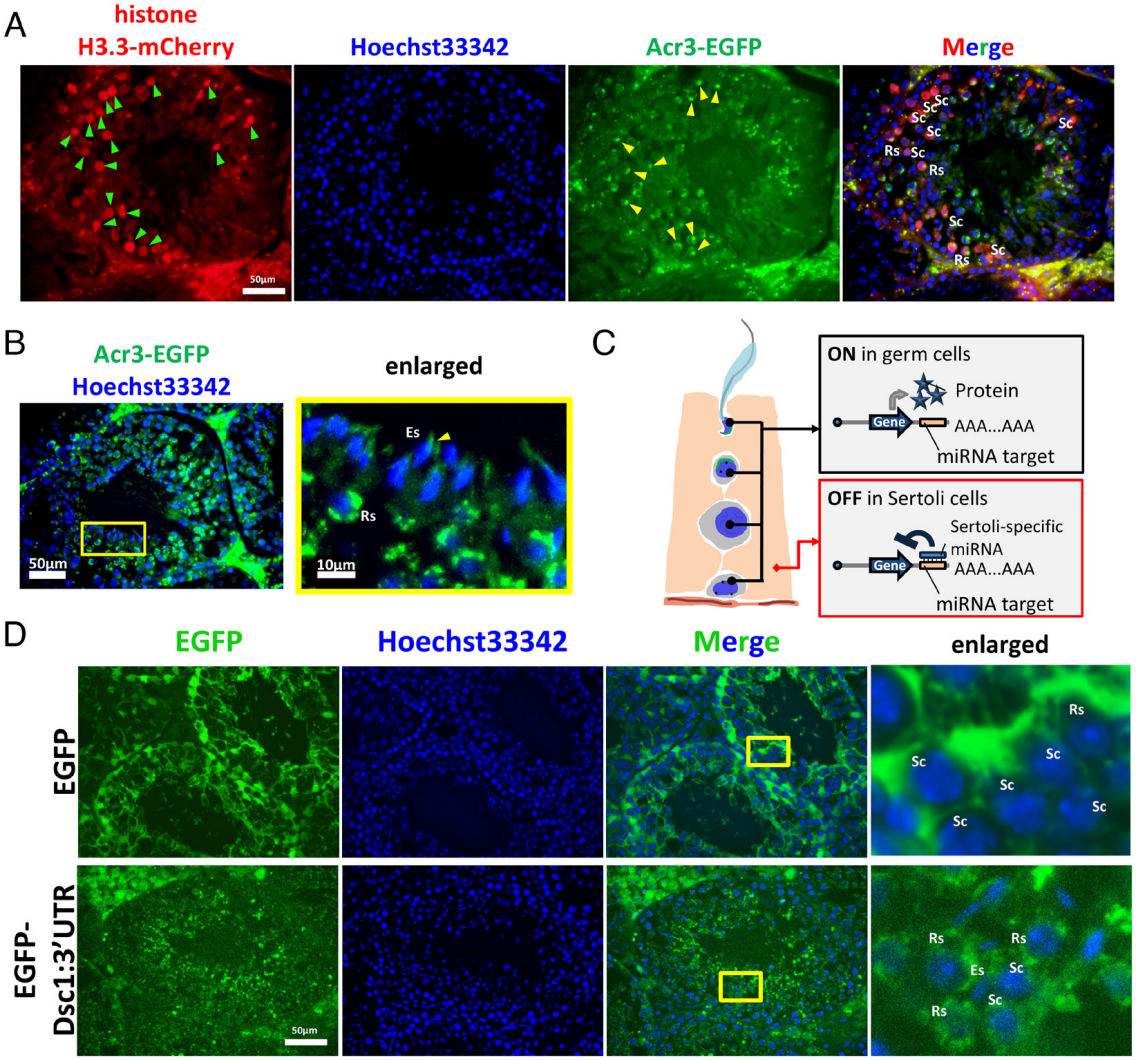
Germ cell-specific expression strategy. (*A*) Fluorescence images of testis sections after LNP-mediated delivery of histone H3.3-mCherry mRNA (Green arrowheads) and *Acr3*-*EGFP* mRNA (Yellow arrowheads). Sc: Spermatocyte, Rs: Round Spermatid. (*B*) Observation of the acrosome after LNP-mediated delivery of *Acr3*-*EGFP* mRNA. The yellow box shows the enlarged image. (*C*) Schematic illustration of the strategy for germ cell-specific expression using miRNA. (*D*) Comparison of fluorescence expression with or without the *miR-471* recognition sequence.

Next, to achieve subcellular localization of target proteins, we designed mRNA constructs encoding EGFP targeted to the acrosome (Acr3-EGFP) and mCherry localized to the nucleus (histone H3.3-mCherry). These mRNAs were encapsulated in LNPs and introduced into wild-type seminiferous tubules via the rete testis. In contrast to EGFP and mScarlet signals, Acr3-EGFP and H3.3-mCherry signals were enriched in spermatogenic cells, and their signals were localized to the acrosomes and nuclei of spermatocytes, respectively (Fig. 2*A*). In addition, Acr3-EGFP signals were detected in the acrosomes of elongated spermatozoa, as early as one day after injection (Fig. 2*B*). These results confirmed successful LNP-mediated mRNA delivery to meiotic and postmeiotic germ cells.

### Spermatogenic Cell-Biased Expression Using MicroRNA Target Sequences.

The cell-type specificity of expression is important because of the unknown impact of ectopic expression in other cell types. To circumvent this issue and achieve germ cell-specific expression, we designed an mRNA construct that suppresses expression in Sertoli cells via microRNA-mediated silencing (Fig. 2*C*). Specifically, the 3′ untranslated region (UTR) of desmocollin 1 (*Dsc1*), a validated target of *miR-471*, highly expressed in Sertoli cells (20), was inserted into the 3′-UTR of *EGFP* mRNA. LNPs containing this mRNA (LNP-*EGFP*-*Dsc1*:3′UTR) were introduced via the rete testis, and their expression patterns were analyzed. The results showed that expression was biased toward germ cells in seminiferous tubules (Fig. 2*D*).

### Restoration of Spermatogenesis in *Pdha2* Knockout Mice.

**Fig. 3.**
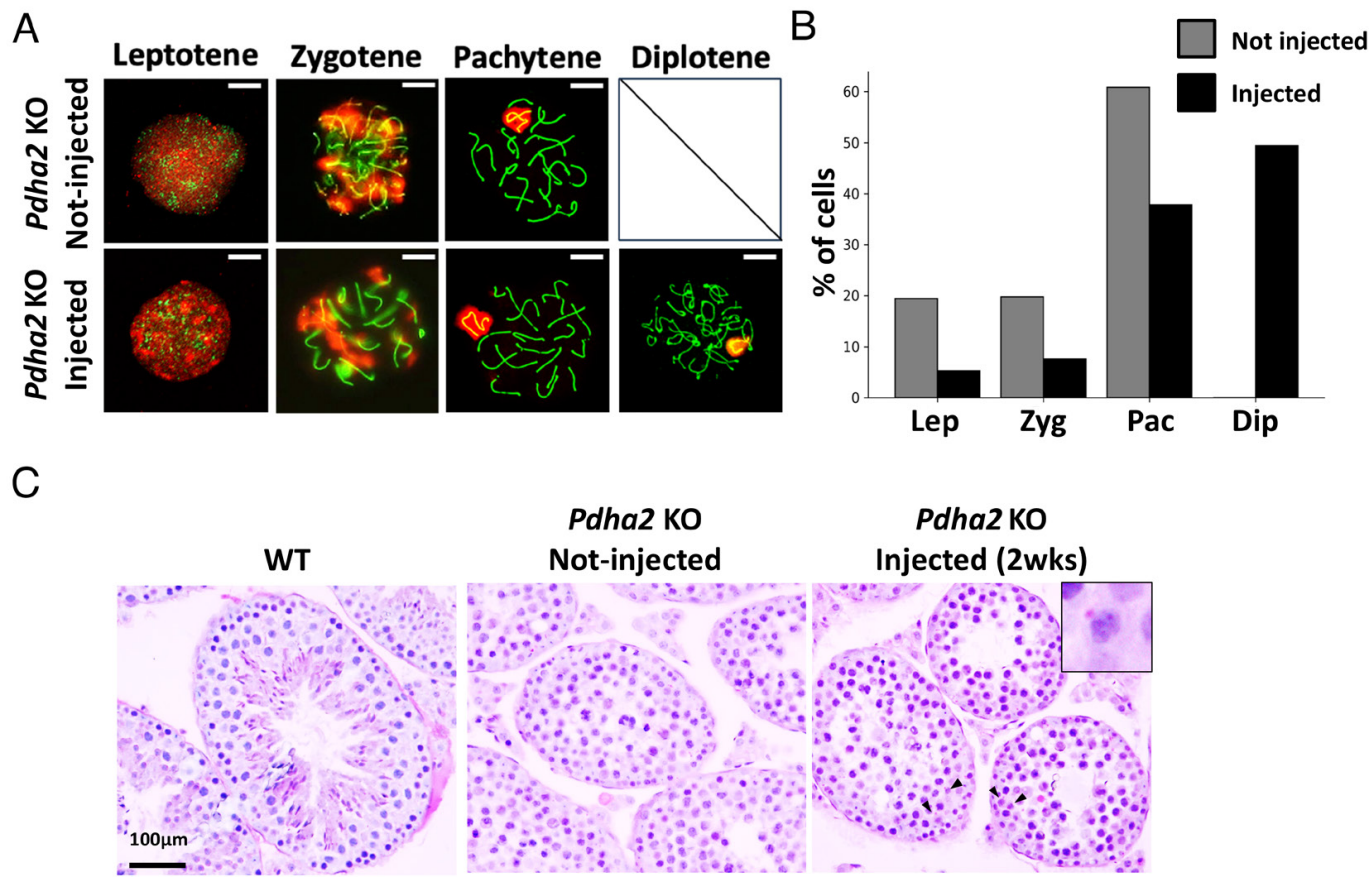
Rescue of *Pdha2* KO Testes. (*A*) Observation of chromosome spreads. Green indicates SCP3, and red indicates γH2AX. The spread nuclei of prophase spermatocytes collected from adult *Pdha2*^−/−^ and injected *Pdha2*^−/−^ male mice were stained with anti-SYCP3 (green) and γH2AX (red) antibodies. XY indicates the sex chromosomes encircled by γH2AX signal during the pachytene and diplotene stages. (Scale bar, 10 µm.) Experiments were repeated at least twice with consistent results. (*B*) Graph showing the proportion of Prophase I stages. (*C*) Image of testis sections stained with H-PAS.

The *Pdha2* KO mouse model, which exhibits severe NOA due to meiotic arrest at the pachytene stage (14), was used to assess the therapeutic efficacy of LNP-mediated mRNA replacement therapy in restoring spermatogenesis. LNPs containing *Pdha2* mRNA with the 3’ UTR of Dsc1 (LNP-*Pdha2*-*Dsc1*:3′UTR) were administered in vivo, and the mice were monitored for several weeks. While chromosome spread analysis at 2 wk postinjection (wpi) revealed that the noninjected group was arrested at the pachytene stage, the injected group exhibited progression to the late diplotene stage (Fig. 3 *A* and *B*). Histological examination of Hematoxylin-Periodic acid–Schiff (H-PAS)-stained testes from the KO mice showed an absence of round spermatids in the noninjected group, whereas the injected group revealed the presence of round spermatids (Fig. 3*C*). PNA (Peanut agglutinin) positive spermatids were found in about a half of the seminiferous tubules while none of the tubules were positive in untreated testis (47.4 ± 9.3%; see *SI Appendix,* Fig. S4 *A* and *B*). This is consistent with the EGFP expression rate of 54.9% in the testis injected with LNP-*EGFP* (*SI Appendix*, Fig. S1).

### Viable Offspring Production Via TESE-ICSI.

**Fig. 4.**
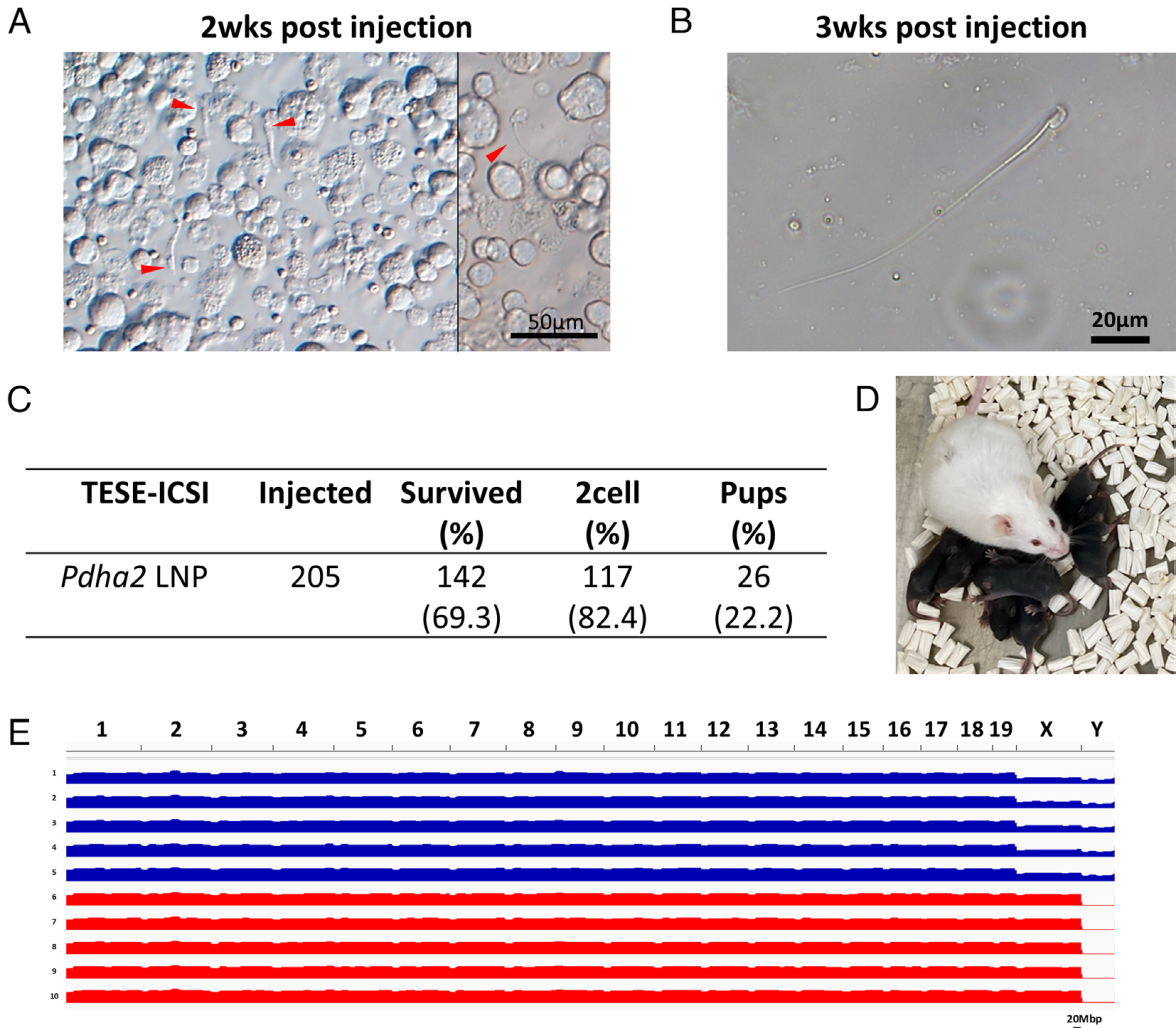
Acquisition of offspring by TESE-ICSI. (*A*) Representative image of testicular cells at 2 wk postinjection (2 wpi). (*B*) Sperm image at 3 wpi. (*C*) Results of TESE-ICSI. (*D*) Image of the obtained offspring. (*E*) Genome-wide visualization of lcWGS data using Integrative Genomics Viewer (IGV).

At two wpi, the tunica albuginea was removed from the *Pdha2* KO testes injected with LNP-*Pdha2*-*Dsc1*:3′UTR, and microscopic examination of dissociated testicular cells revealed elongated spermatids corresponding to steps 11 to 13 of spermiogenesis (Fig. 4*A*). By three wpi, more advanced spermatid stages, including steps 14 to 16, were observed (Fig. 4*B*; see *SI Appendix,* Fig. S5*A*). ICSI using testicular spermatozoa collected from 3-wpi testes resulted in the production of viable offspring, with 26 pups obtained from 117 two-cell embryos (22.2%) (Fig. 4 *C* and *D*). Genotyping confirmed that all offspring were heterozygous for the *Pdha2* mutant allele (*SI Appendix,* Fig. S5*B*). In addition, we confirmed that the introduced mRNA was not reverse-transcribed and inserted into the genome (*SI Appendix,* Fig. S5*C*). Offspring developed normally for 60 d (*SI Appendix,* Fig. S5*D*) without gross morphological or developmental abnormalities. Whole-genome sequencing of five male and five female offspring confirmed the absence of large-scale deletions or insertions (>1 Mbp) by copy number variation analysis (Fig. 4*E*; see *SI Appendix,* Fig. S6). To assess their fertility, male offspring (F1) mice were then mated to females and successfully sired F2 offspring (*SI Appendix,* Fig. S7).

## Discussion

In the present study, we demonstrated that LNP injection into the rete testis enables efficient mRNA delivery to both male germ cells and Sertoli cells. To achieve selective expression in spermatogenic cells, we incorporated the 3′-UTR of *Dsc1*, which contains a binding site for the Sertoli cell-specific microRNA *miR-471.* The presence of the 3′-UTR of *Dsc1* switched off the mRNA expression in Sertoli cells, resulting in preferential expression in germ cells. This strategy was applied to the delivery of *Pdha2* mRNA, and its supplementation in *Pdha2* KO mice successfully restored spermatogenesis. The use of ICSI with testicular spermatozoa from these injected *Pdha2* KO mice resulted in the birth of offspring. Offspring were healthy and fertile without apparent abnormalities, and genomic analysis revealed no detectable genomic alterations.

Targeted delivery and expression control at the cellular level are essential for developing effective mRNA-based therapies. Although we did not investigate lipid composition-based specificity in this study, the physicochemical properties of LNPs—determined by the types and ratios of lipid components such as ionizable lipids, phospholipids, cholesterol, and PEG-lipids—may influence cellular uptake, endosomal escape efficiency, and tissue tropism (21). In particular, modifications to lipid composition, such as adjusting the structure or proportion of ionizable lipids, or replacing PEG-lipids with cleavable or biodegradable alternatives, may enhance delivery specificity to target cells or tissues. These strategies may therefore prove valuable in future applications to improve the spatiotemporal precision of mRNA delivery.

In addition to cell-specific specificity of the delivery of the mRNA, posttranscriptional regulation, including mRNA stability and translational control, is an essential factor influencing expression outcomes. One promising approach involves incorporating miRNA target sequences to achieve cell-specific suppression. This strategy is beneficial for turning off expression in off-target cells after nonspecific uptake of LNPs. In our study, we introduced a degradation switch by adding the 3’-UTR of *Dsc1*, a known target of *miR-471*, to the mRNA sequence (20). This approach allowed selective degradation in Sertoli cells (Fig. 2 *C* and *D*). While *miR-471* is effective in mouse testis, the evaluation of human Sertoli cell-specific miRNAs (e.g., *miR-202-5p*; (22)) will be crucial for clinical translation of our approach. Screening and validating such miRNAs could enable the development of delivery systems that favor expression in human germ cells while avoiding somatic cell expression. An alternative strategy could involve the use of stabilizing elements downstream of the polyA tail, which can activate translation upon miRNA-mediated cleavage specifically in germ cells (23). This ON-switch mechanism could be effectively combined with miRNA-mediated suppression in Sertoli cells, offering complementary control over cell-specific expression.

Regulation at the protein level may also represent a promising avenue for further investigation. One potential strategy involves the incorporation of degron sequences (24), which can target the encoded protein for rapid ubiquitin-mediated degradation in specific cellular contexts. By linking degrons to mRNA-encoded proteins, it may be possible to achieve posttranslational suppression of expression in nongerm cells, assuming appropriate cell-type-specific E3 ligases are active. This alternative approach could provide an additional layer of control, particularly when transcriptional or translational specificity is insufficient. Moreover, subcellular targeting using organelle-specific localization signals is also effective. In the present study, we investigated the feasibility of delivering mRNA into germ cells by injecting LNPs carrying Acr3-EGFP and histone H3.3-mCherry constructs (Fig. 2 *A* and *B*). The observed localization to the acrosome and spermatocyte nuclei is attributed to the signal peptide of acrosin (19) and the meiotic replacement of histone H3.1/H3.2 with H3.3 during sex chromosome inactivation (25). These results suggest that targeting germ cells may benefit from the intrinsic properties of sperm-specific proteins and processes. Given the unique cellular characteristics of sperm, a combinatorial approach incorporating multiple levels of regulation—ranging from mRNA delivery and transcription to translation and protein stability—may be beneficial for infertile men. Such mechanisms could further refine the spatial and temporal specificity of mRNA-based interventions.

A previous study reported that LNPs containing self-amplifying RNA generated motile epididymal spermatozoa within 10 d in *Dmc1* KO mice with meiotic arrest (26), but this is not considered physiologically possible. Spermiogenesis after meiosis takes around 2 wk in the testis, and sperm maturation takes around 1 wk in the epididymis (27). Here, we used *Pdha2* KO mice in which spermatogenesis also arrests at the pachytene stage (Fig. 3). We uncovered round spermatids after 2 wk (Fig. 4*A*) and spermatozoa after 3 wk of treatment (Fig. 4*B*), consistent with the normal physiological schedule of spermatogenesis in mice. Since ICSI using testicular spermatozoa eliminates the need for sperm maturation, we directly performed ICSI with testicular spermatozoa and successfully obtained offspring (Fig. 4 *C* and *D*). In the clinical context, microTESE-based retrieval of testicular spermatozoa followed by ICSI is a well-established and feasible approach, supporting the applicability of our method.

Because LNP treatment successfully restored spermatogenesis in *Pdha2* KO mice, evaluating the resulting genome integrity was essential to confirm the absence of chromosomal rearrangements and deletions. Whole-genome sequencing confirmed the absence of large-scale deletions, indicating that mRNA supplementation via LNP can generate healthy offspring without inducing detectable genomic alterations (Fig. 4*E*). Cell-based methods, such as extracellular vesicles (28) and viral vectors, may carry a risk of contamination due to their cellular origin. In contrast, LNPs are fully synthetic and composed of chemically defined components. This property minimizes the risk of introducing foreign genomic material and enhances the safety and reproducibility of the delivery method, further supporting its clinical applicability. These considerations highlight the therapeutic potential of LNP-based delivery for restoring spermatogenesis in NOA in the clinic.

A current limitation of our approach is the requirement for in vivo administration to the testis. However, future integration with seminiferous tubule culture techniques (29, 30) may enable ex vivo treatment. In this alternative approach, surgically retrieved seminiferous tubule segments could be treated by injecting LNPs directly into the seminiferous tubules, allowing for controlled mRNA delivery and enhancing safety by avoiding direct in vivo exposure. Since TESE-ICSI is a clinically established method that requires limited spermatozoa, even partial restoration of spermatogenesis through ex vivo treatment could be sufficient for successful fertilization.

In conclusion, mRNA replacement via LNPs restored spermatogenesis, enabled viable offspring production in a genetic model of azoospermia, and did not cause any observable genomic disruptions. Our results highlight the therapeutic potential of LNP-mediated gene supplementation for the treatment of NOA in men.

## Materials and Methods

### Animals.

Our studies adhered to the ARRIVE guidelines 2.0 and the Guide for the Care and Use of Laboratory Animals. All mouse experiments were approved by the Animal Care and Use Committee of the Research Institute for Microbial Diseases at Osaka University (#Biken-AP-H30-01). Mice were obtained from Japan SLC Inc. (Shizuoka, JP) and bred in a specific pathogen-free environment as described (31). GBGS Tg mice (32) and *Pdha2* KO mice (14) have been previously reported. The genetically modified mice (33) can be obtained from the RIKEN BioResource Research Center in Ibaraki, Japan, or the Center for Animal Resources and Development at Kumamoto University, Japan.

### Plasmid Generation.

To generate pCAG-EGFP-Dsc1:3’UTR, the pCAG-EGFP plasmid (33) was digested with HindIII and NotI, and the two oligos containing Dsc1:3’UTR were annealed and ligated into the plasmid using ligase (see primer information in the *SI Appendix* Table).

### RNA Preparation.

*EGFP* mRNA was purchased from TriLink BioTechnologies Inc. (L7601; San Diego, CA). For *Acr3-EGFP* mRNA, pAcr-Acr3-EGFP (19) was used as a template. PCR was performed at 94 °C, 60 °C, and 72 °C for 40 cycles. mRNA was synthesized using the mMESSAGE mMACHINE T7 Transcription Kit (Thermo Fisher Scientific, Waltham, MA). For *EGFP-Dsc1*:3’UTR mRNA, pCAG-EGFP-Dsc1:3’UTR was used as a template. For *Pdha2*-*Dsc1*:3’UTR, pClgn-Pdha2-PA-1D4 was used as the template. For *mScarlet* mRNA, pmScarlet_C1 (Addgene #85042; 19) was used as a template. Primer information is provided in the *SI Appendix* Table.

### LNP Preparation.

Lipids, including SS-OP (10 mM) (COATSOME SS-OP, NOF corporation, Kawasaki, JP), DOPC (10 mM) (COATSOME MC-8181, NOF corporation), cholesterol (10 mM) (C8667-500MG, Sigma Aldrich, MO), and DMG-PEG2000 (2 mM) (SUNBRIGHT GM-020, NOF corporation, Kawasaki, JP), were prepared in ethanol. The mRNA encoding EGFP (L-7601, TriLink, San Diego, CA) or synthesized RNA was diluted to 6.56 mg/mL of concentration in 20 mM citric acid solution (pH 5.0). These lipid and mRNA solutions were mixed using NanoAssemblr Ignite (total flow rate: 12 mL/min; flow rate ratio: water/ethanol = 3/1 (v/v), Precision NanoSystems Inc., Vancouver, BC, CA). 20 mM MES buffer (pH 6.0) was added to the mixture. Then, the solution was replaced with TBS (pH 7.4) by ultrafiltration with Amicon Ultra-4 (UFC810096, Merck, KGaA, Germany). The mRNA concentration encapsulated into LNP was measured by RiboGreen assay (Quant-iT RiboGreen RNA Assay Kit, R11490, Invitrogen, Carlsbad, CA).

### Rete Testis Injection.

We used 6 to 8-wk-old male mice for these studies. Rete testis injection was performed using a previously reported method (17) with slight modifications. The injection needle was prepared from borosilicate glass (B100-75-10, Sutter Instrument, Novato, CA) using a micropipette puller (MODEL P-1000IVF; heat 770, pull 10, vel 50, delay 200, pressure 500; Sutter Instrument). The tip diameter was further adjusted by slightly breaking the glass needle at the tip during injection.

### Immunofluorescent Staining Using Chromosome Spreads From Mouse Spermatocytes.

Testis without membrane was incubated with hypotonic buffer (30 mM Tris [pH 8.2], 50 mM sucrose, 17 mM trisodium citrate dihydrate, 5 mM EDTA [pH 8.0], 2.5 mM dithiothreitol, 0.5 mM phenylmethylsulfonyl fluoride) for 40 min at RT after tunica albuginea removal. The samples were then transferred to 100 mM sucrose, and germ cells were released by gently squeezing the seminiferous tubules. Germ cells were fixed in 1% PFA with 0.15% Triton X-100. The germ cell suspension was evenly spread on the slides, followed by incubation in a humid chamber for 2 h at RT and then washed in 0.04% PhotoFlo solution in PBS to remove salts (Kodak Alaris, Rochester, NY). For immunofluorescence, chromosome spreads on slides were blocked with 3% nonfat milk in PBS for 10 min, then incubated with primary antibodies (Mouse monoclonal anti-γH2A.X (Millipore Cat# JBW301, Burlington, MA), Rabbit polyclonal anti-SYCP3, Abcam Cat# ab15090, Rabbit polyclonal anti-DDX4, Abcam Cat# ab13840, Cambridge, UK) in 3% nonfat milk at 4 °C overnight. Slides were washed three times in PBS with 0.1% Tween 20 (PBST) and incubated with secondary antibodies for 1 h at RT. The slides were washed three times and cover-slipped with Immu-Mount. Images were captured with an FV3000 confocal microscope (Olympus, Tokyo, JP). The XY bodies were identified as chromosomes with a condensed, asymmetrical SYCP3 configuration with partial synapsis, distinct from linear and fully synapsed autosomes (34).

### Histological Analysis.

Collected testes were fixed in 4% PFA for 6 h. Tissue samples were embedded in Technovit 8100 (Kulzer GmbH, DE) and observed under a BX53 microscope (Olympus). Fluorescence images were captured using a BZ-H4XF system (Keyence Corporation, Osaka, JP). For PAS staining, the sections were oxidized using 0.5% periodic acid (Nacalai Tesque, Kyoto, JP) for 10 min at room temperature, followed by incubation in Schiff reagent (Sigma-Aldrich) for 15 min. The slides were then washed under running tap water for 5 min to remove excess reagents and enhance color development. Counterstaining was performed using Mayer’s hematoxylin (FUJIFILM Wako Pure Chemical Corporation, Osaka, JP; 131-09665) for approximately 30 s to 1 min.

### ICSI.

Hormone treatment of mice, cumulus-oocyte complex collection and processing, sperm head preparation and ICSI, embryo incubation and transfer to the oviducts of pseudopregnant ICR females, and delivery of offspring via Caesarean section or naturally were performed as described (29).

### Genotyping.

The PCRs for detecting the wild-type and knockout alleles of *Pdha2*, as well as for confirming that the mRNA introduced via LNP was not reverse-transcribed and integrated into the genome, were performed with the following cycling conditions: 94 °C for denaturation, 60 °C for annealing, and 72 °C for extension, for a total of 40 cycles. Primer sequences are listed in the *SI Appendix* Table.

### DNA Extraction, Library Preparation, and Whole-Genome Sequencing.

Genomic DNA was extracted from mouse digit tissues. Briefly, excised digits were immersed in 160 μL of phosphate-buffered saline (PBS) and mechanically disrupted using a TissueLyser II system (QIAGEN, Hilden, DE) with a 5 mm zirconia bead. Following tissue homogenization, genomic DNA was purified using the QIAamp DNA Mini Kit (QIAGEN) according to the manufacturer’s protocol. DNA libraries were prepared using the MGI FS PCR-Free DNA Library Prep Kit (MGI Tech, Shenzhen, CN), and sequencing was performed on a DNBSEQ-G400RS platform (MGI Tech). Paired-end 150 bp reads were generated, yielding an average genome coverage of approximately 10× per sample. The data have been deposited in DDBJ under the accession number PRJDB35531.

### Read Processing and Copy Number Analysis.

Sequencing reads were trimmed to remove adapter sequences using cutadapt (v3.2), and the resulting high-quality reads were aligned to the mouse reference genome (GRCm39) using bowtie2 (v2.3.5.1) with default parameters. Aligned reads were converted to BAM format and sorted using SAMtools (v1.20). To assess potential large-scale genomic alterations such as insertions or deletions, genome-wide read depth profiles were generated using bamCompare from the deepTools suite (v3.4.3). Log_2_ fold changes in read depth between samples were calculated at 1 Mb bin resolution, providing an overview of genomic structural consistency across samples.

## Supplementary Material

Appendix 01 (PDF)

## Data Availability

fastq data have been deposited in DDBJ (PRJDB35531) (35).
