# Supplementary material for: Sperm and offspring production in a nonobstructive azoospermia mouse model via testicular mRNA delivery using lipid nanoparticles
Source: Proc Natl Acad Sci U S A. 2025 Oct 13;122(42):e2516573122. doi: 10.1073/pnas.2516573122 (PMC12557808; doi:10.1073/pnas.2516573122)
Supplement: Supplementary file 1 — Appendix 01 (PDF) [file pnas.2516573122.sapp.pdf]

PNAS

Supporting Information for
Spermatozoa and offspring production in a non-obstructive azoospermia mouse model via testicular mRNA delivery using lipid nanoparticles

Authors
Daisuke Mashiko, Chihiro Emori, Yuki Hatanaka, Daisuke Motooka, Chen Pan, Yuki Kaneda, Martin M. Matzuk, and Masahito Ikawa

Correspondence to mmatzuk@bcm.edu or ikawa@biken.osaka-u.ac.jp

This PDF file includes:

Appendix figures S1 to S7
Appendix table

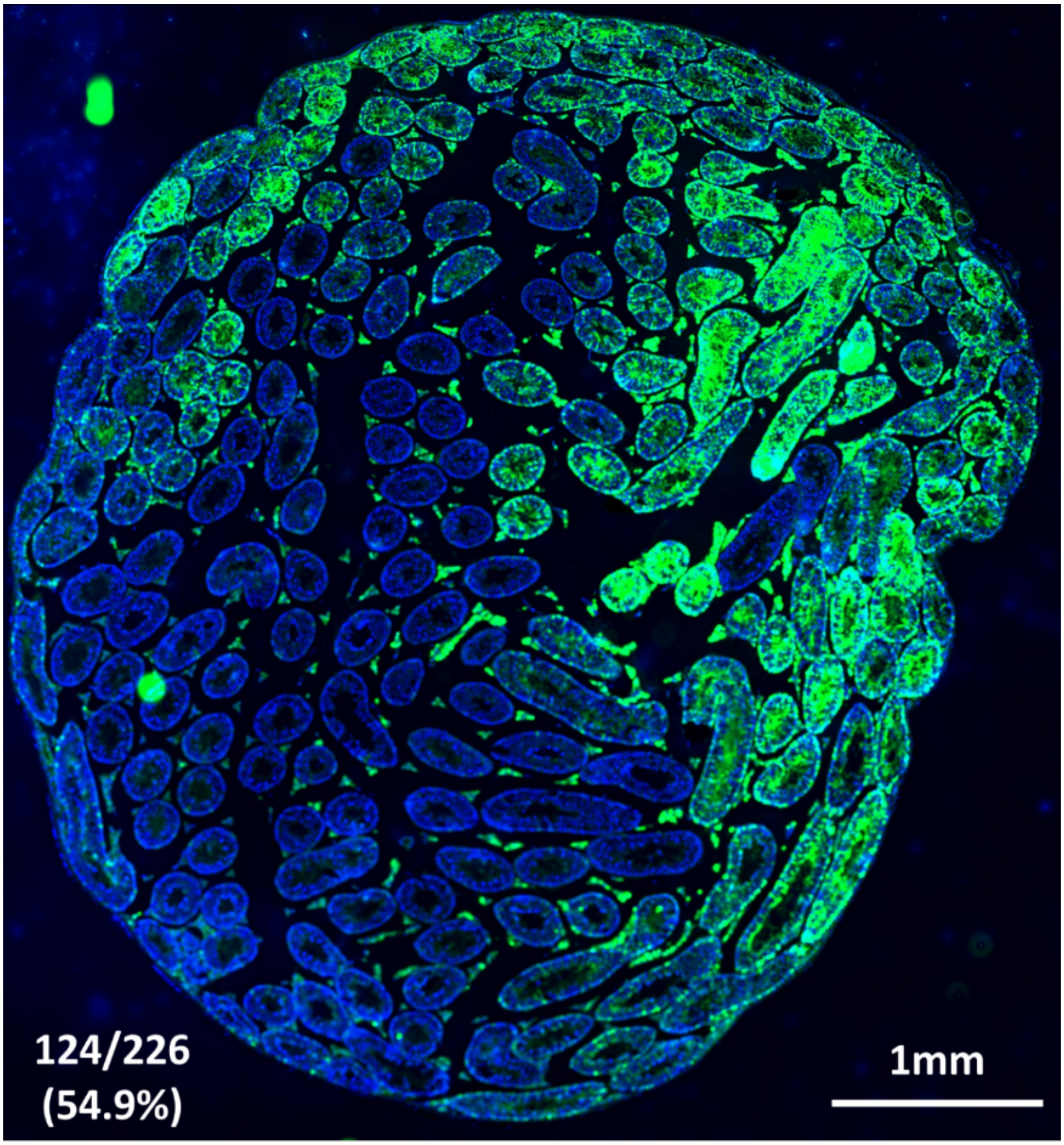

fig. S1. Whole image of testis sections after LNP-EGFP injection into seminiferous tubules. The whole image of the testis was captured using a Keyence fluorescence microscope. The volume of the injected solution was approximately 15 µL.

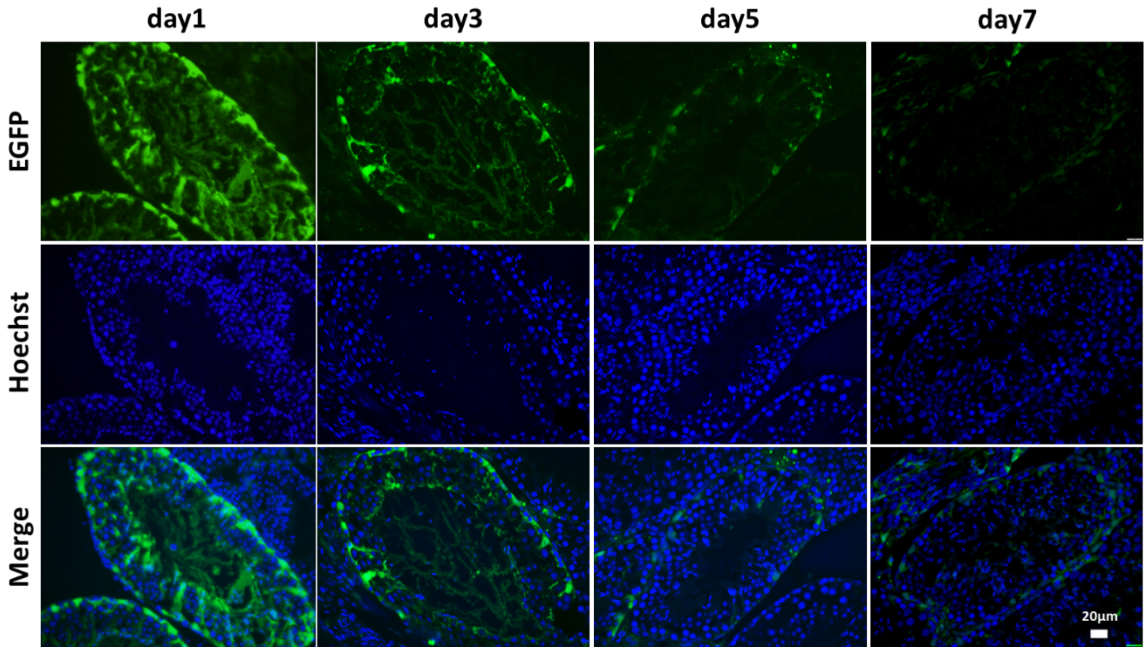

fig. S2. mRNA delivery into testicular cells using LNPs. (A) To examine the duration of fluorescence, mice were sacrificed on days 1, 3, 5, and 7 after LNP-EGFP injection, and tissue sections were prepared for fluorescence observation.

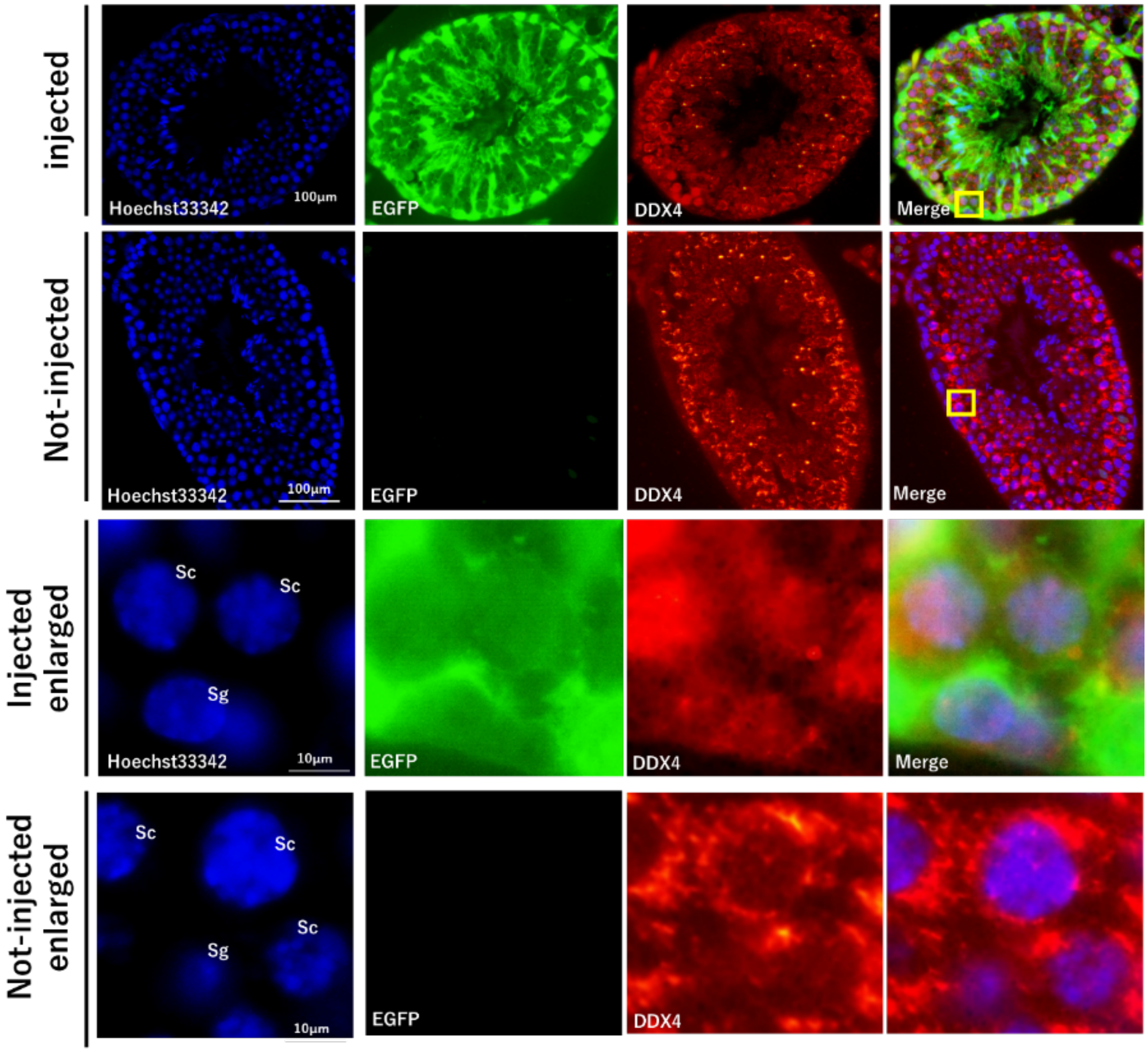

fig. S3. mRNA delivery into spermatogenic cells using LNPs. To detect the EGFP signal, images were acquired under 488 nm excitation with identical exposure settings. Anti-DDX4 antibody (ab13840, Abcam, UK) was incubated for 1 hour, followed by a 1-hour incubation with a secondary antibody (rabbit anti-IgG conjugated with Alexa Fluor 568, Thermo Fisher Scientific, USA). Sg: Spermatogonia, Sc: Spermatocyte.

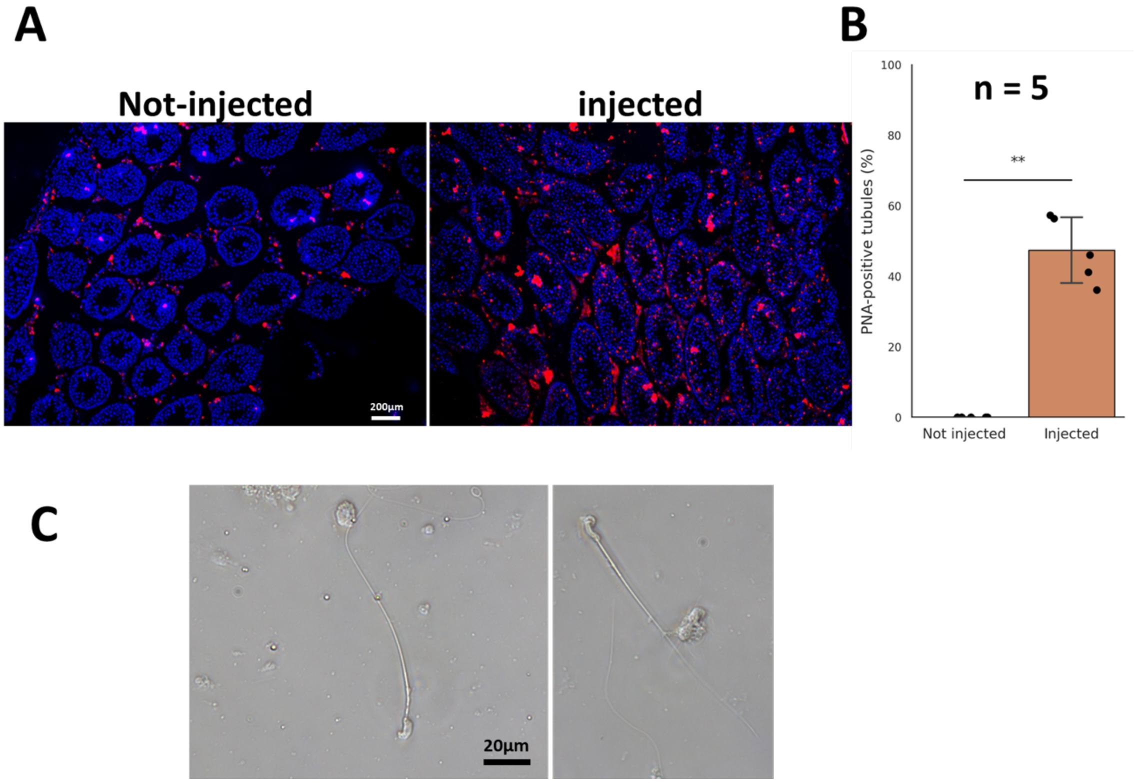

fig. S4. LNP-*Pdha2* mediated recovery of spermatogenesis in *Pdha2* KO testes. (A) The image shows PNA staining of testis sections. (B) Graph showing the proportion of PNA-positive seminiferous tubules at 2 weeks post-injection; Wilcoxon rank-sum test, *P* = 0.0075. (C) Sperm obtained from *Pdha2* KO mice after three weeks of LNP-*Pdha2* injection.

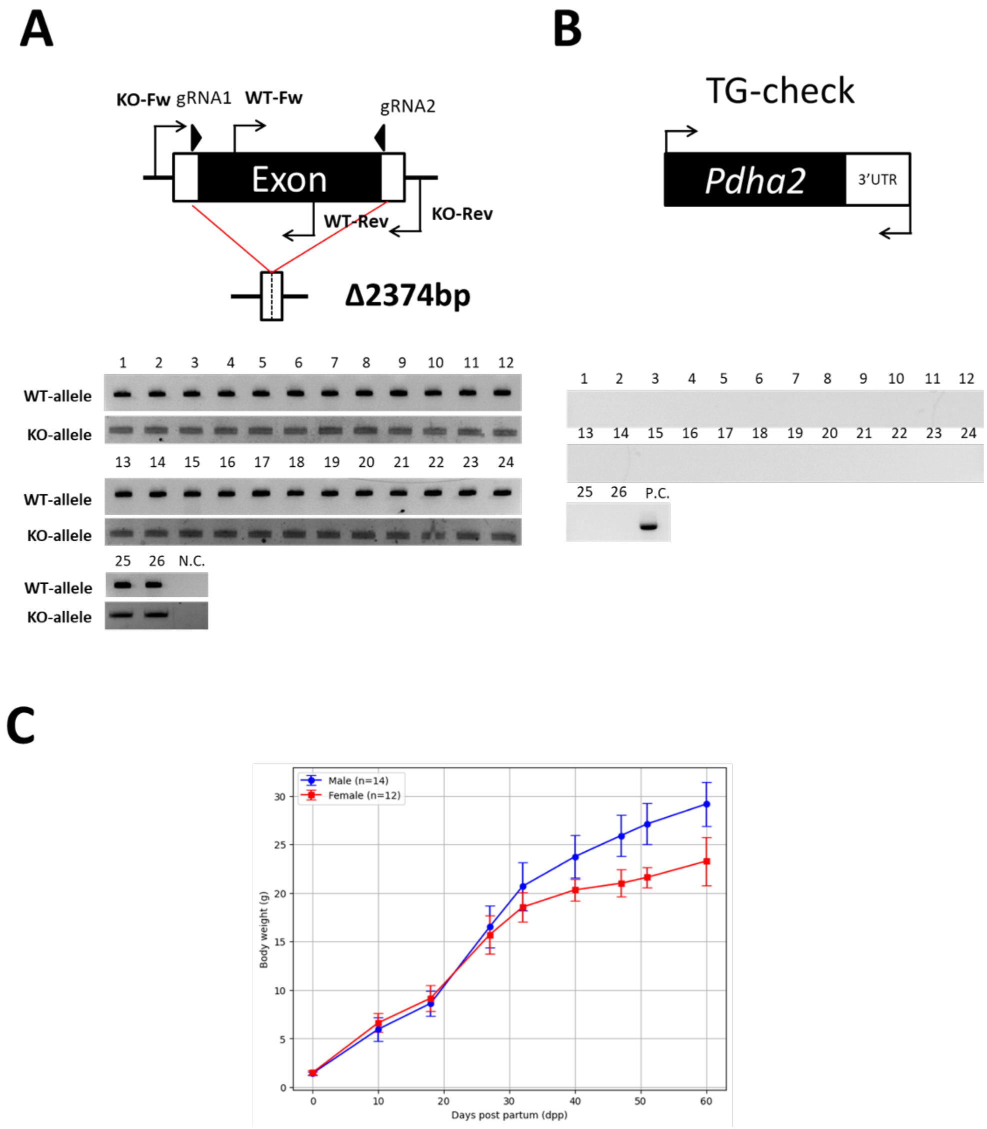

fig. S5. Offspring obtained from Pdha2 KO male mice treated with LNP-*Pdha2*. (A) PCR detection of *Pdha2* alleles in offspring. (B) PCR detection of the *Pdha2* transgene. (C) Growth curve of pups obtained by TESE-ICSI. Blue, male; Red, female.

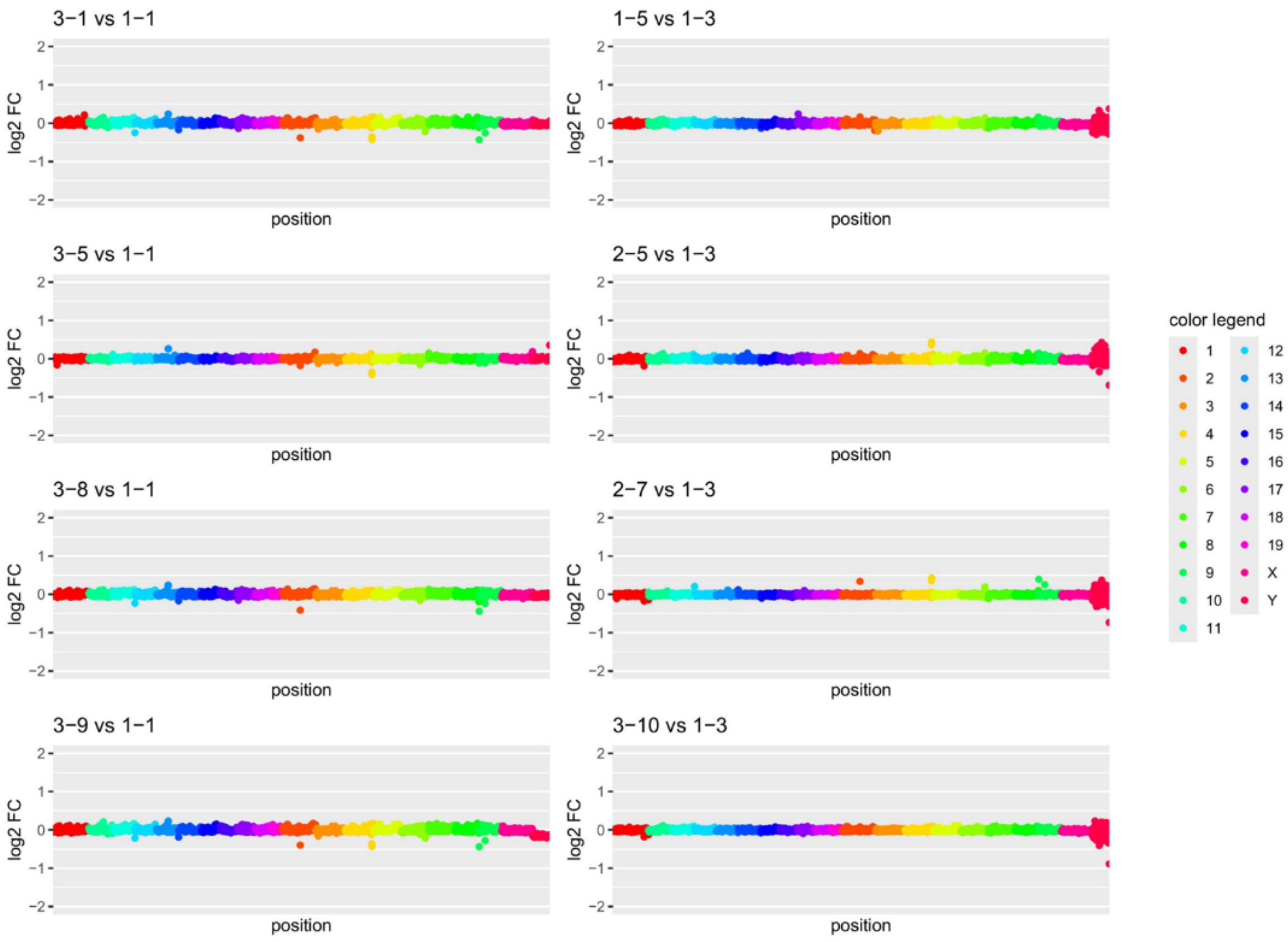

fig. S6. Copy Number Variation analysis of offspring obtained by LNP-*Pdha2* injection into *Pdha2* KO testis. The left panels show the males, and the right panels show the females. Each is compared to a randomly selected control individual. The binning size is 1 Mbp.

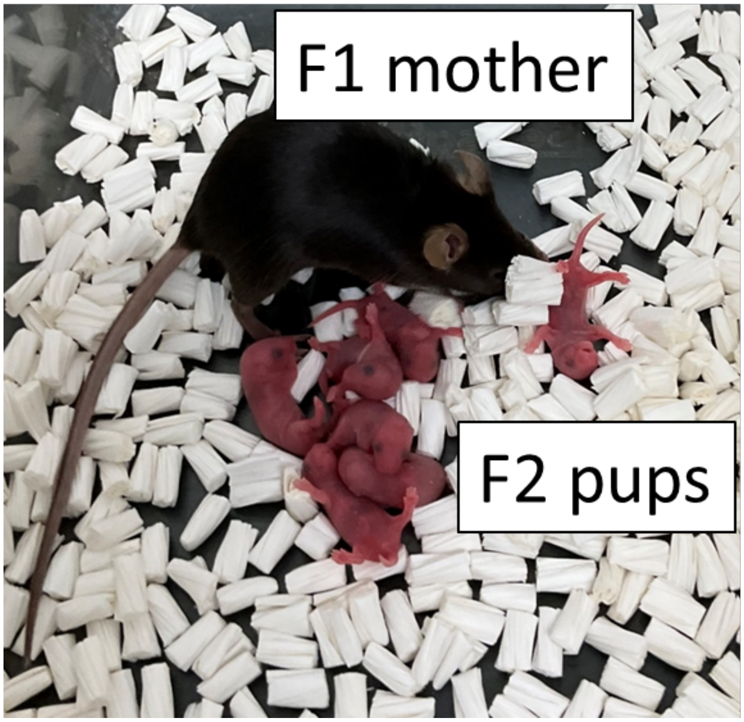

fig. S7. Fertility of F1 mother derived from *Pdha2* KO male mice. The picture is of an F1 mother and her F2 offspring obtained by natural mating.

Appendix table. Primer list.

To generate pCAG-EGFP-3'UTR (DSC1)

oligo#1	agcttAGTACTaacACAAGCATTGTGAAGACATACGTgc
oligo#2	ggccgcACGTATGTCTTCACAATGCTTGTgttAGTACTa

For mRNA

Acr3-EGFP-Fw	TAATACGACTCACTATAGGGAGAgccgccATGCTGCCAACTGTCGCTG
Acr3-EGFP-Rev	TGAGAGACACAAAAAATTCCAACACACTATTGCAA
EGFP-3'UTR (Dsc1)-Fw	TAATACGACTCACTATAGGGAGAgccgccATGGTGAGCAAGGGCGAGG
EGFP-3'UTR (Dsc1)-Rev	TGAGAGACACAAAAAATTCCAACACACTATTGCAA
Pdha2-Fw	TAATACGACTCACTATAGGGAGAgccgccATGAGGAAAATGCTGACCGCTG
Pdha2-Rev	ACGTATGTCTTCACAATGCTTGTgttCTAACTGTGGGACTTATACTTGAGCCAC
mScarlet-Fw	TAATACGACTCACTATAGGGAGAgccgccATGGATAGCACCGAGGCAGT
mScarlet-Rev	TGAGAGACACAAAAAATTCCAACACACTATTGCAA

genotype

TG-detection-Fw	ATGAGGAAAATGCTGACCGC
TG-detection-Rev	gttCTAACTGTGGGACTTATACTTGAGCCAC
KO-Fw	aaaacaaccagaggaatggaagg
KO-Rev	Gagcatctctccaagtccct
WT-Fw	gctctcagaggactgctgtc
WT-Rev	catcgccgtacaaagccaag
